# Non-monetary numeraires: Varying the payment vehicle in a choice experiment for health interventions in Uganda

**DOI:** 10.1016/j.ecolecon.2019.106569

**Published:** 2020-04

**Authors:** Keila Meginnis, Nick Hanley, Lazaaro Mujumbusi, Poppy H.L. Lamberton

**Affiliations:** a University of Glasgow, United Kingdom of Great Britain and Northern Ireland; b Medical Research Council/Uganda Virus Research Institute & London School of Hygiene and Tropical Medicine, Uganda Research Unit, Uganda; c Economics Division, University of Stirling Management School, Stirling, UK

**Keywords:** Discrete choice experiment, Schistosomiasis, *Schistosoma mansoni*, WASH, Non-monetary numeraires, Shadow wage rate, One health approach

## Abstract

Schistosomiasis is a serious health problem in many parts of Africa which is linked to poor water quality and limited sanitation resources. We administered a discrete choice experiment on water access and health education in rural Uganda, focussing on interventions designed to reduce cases of the disease. Unlike previous studies, we included a payment vehicle of both labour hours supplied per week and money paid per month within each choice set. We were thus able to elicit both willingness to pay and willingness to work for alternative interventions. Respondents exhibit high demand for new water sources. From the random parameter model, only households with knowledge about water-borne parasites are price sensitive and exhibit willingness to pay values. Through a latent class model specification, higher income respondents exhibit higher willingness to pay values for all programme attributes; however, lower income participants have higher willingness to work values for certain new water sources. We found a shadow wage rate of labour that is between 15 and 55% of the market wage rate.

## 1 Introduction

Globally, over 240 million people in low and middle-income countries are infected with the disease schistosomiasis, with over 90% of these infections occurring in sub-Saharan Africa (Sanya et al., 2017). Infections, driven by poor water access and inadequate sanitary resources, are particularly prevalent among children. The costs of the disease include reduced physical and cognitive development in children and long-term severe morbidity, exacerbating the poverty cycle. In low and middle income countries (LMICs), environmental and public health interventions often fail when communities do not see a need for interventions, or do not perceive ownership over these interventions (Sawyer et al., 1998). This situation can occur when communities are not involved in the decision and planning process, the implementation of interventions, and/or the ongoing maintenance of assets provided in the intervention. Interventions which fail to reflect community preferences are unlikely to be effective in the long term. In the case of schistosomiasis, such interventions need to break the cycle of re-infection by improving access to water resources which are un-contaminated by the parasite responsible, and by reducing the rate at which the parasite is re-introduced to the local environment through investments in sanitary resources.

Economists routinely measure people's values for alternative (public) health interventions using stated preference approaches, since these are capable of reflecting both the direction of people's support for alternatives, and how strongly they support or oppose these alternatives (Soekhai et al., 2019). Moreover, stated preference methods allow for monetary evaluation of the benefits and costs of alternative interventions, so that cost-benefit analysis methods can be used to determine socially preferred choices (Carolus et al., 2018; Hanley and Barbier, 2009; Johnston et al., 2017). Discrete Choice Experiments (DCE) are an especially useful method in this regard, since they permit the researcher to evaluate how individual policy “attributes” – such as which specific interventions are employed to achieve a given objective – compare in terms of people's Willingness to Pay, which allows the relative economic value of alternative interventions to be established. Moreover, how the benefits of a specific intervention are valued differently by different groups of people (preference heterogeneity) can also be quantified using this approach. Set against these advantages, DCE approaches have a number of limitations: value estimates are conditional on the exact description of the choice alternatives offered to participants; stated choices may not always be a robust predictor of actual choices dependent on how choices are elicited; and respondents beliefs on the consequentiality of their responses may vary, causing changes in their responses (Johnston et al., 2017).

It is standard practice in the choice experiment literature to include a monetary payment vehicle as an attribute, as this is how researchers obtain estimates of willingness to pay for changes in non-monetary attributes for use in cost-benefit analysis. However, a monetary payment vehicle may not be appropriate in communities with very low levels of income, or where trading can be commonly done via barter. In such settings, researchers have argued for the use of non-monetary payment vehicles such as labour time, which yield an alternate measure of how much of a particular numeraire people are willing to give up to benefit from an improvement in some desired attribute such as a reduction in water pollution.

In the study reported in this paper, we administered a discrete choice experiment using two payment vehicles within the same choice set: a monthly monetary fee and a weekly labour contribution.^1^ Through our unique design, individuals needed to make trade-offs between what they say they can afford in terms of time and of ^money^ for reductions in health risks in each choice scenario. We did this by administering a DCE in Uganda to explore willingness to pay and willingness to work for interventions that improve water access and reduce respondents' risk of contracting *Schistosoma mansoni*, the parasite causing intestinal schistosomiasis in these communities.^2^ We surveyed respondents in rural Uganda and asked their preferences for community interventions that improve water access and health education, thus reducing risks of catching schistosomiasis. We included both labour and money as payment vehicles for the interventions. By doing so, we believe that respondents will be more able to make choices over interventions through contributing a combination of both time and money. Additionally, including labour as an attribute could lead to higher levels of ownership over possible interventions, since individuals are committing their own time to help maintain the quality of risk-reducing investments. The comparison of money and labour payment vehicles in discrete choice experiments is not new (e.g. Gibson et al. (2016); Rai and Scarborough, 2015); however, our study is novel in that both payment types are included within each choice set within a public health context. As far as we are aware, this has only been done in two other studies (de Rezende et al., 2015; Rai and Scarborough, 2012), both within ecological contexts.

The remainder of paper is structured as follows. Section 2 reviews the literature on stated preference studies which incorporate the use of alternative payment vehicles. Section 3 describes the field setting and Section 4 the experimental design and econometric framework. Sections 5 presents the model results. Section 6 concludes this paper.

## 2 Literature review: paying with time or paying with money?

This section explores literature that has employed stated preference methodologies in LMICs (Low and Middle-Income Countries) using non-monetary payment vehicles. First, it is important to note that responses to stated preference studies are context dependent: how a respondent answers a DCE will likely vary depending on whether they are being asked about biodiversity conservation, transport investments or interventions targeted at human health. This context-dependence can be due to varying degrees of participant knowledge about the issue(s) over which choices are made (Needham et al., 2018) or different degrees of salience to the respondent (Ghose and Lowengart, 2013). Our study explicitly focused on a human health issue. While not all papers presented in this section are related to human health, they all explore the use of non-monetary numeraires. They are thus relevant to the design of our study, and to its contribution to the broader field of stated preference choice experiments.

Non-monetary payment vehicles have been used in both discrete choice experiments (DCE) and contingent valuation (CV) studies in LMICs. Eom et al. (2006) administered a contingent valuation study to assess willingness to pay or work for water quality improvements to the Man Kyoung River in Korea. They found that levels of willingness to work greatly exceeded “equivalent” values of willingness to pay. These findings are similar to Vásquez (2014) who administered a study on willingness to pay or work for an improved water system in Guatemala. Respondents received a CV choice card with either a monthly fee or a labour contribution required for the improved service. They found that age is the only individual characteristic that significantly affects willingness to pay values, with older respondents less likely to vote in favour of projects that improve water quality. Larger households were significantly more likely to vote in favour of a project, likely given that larger households could distribute the labour hours across household members. The shadow value of labour as implied by these trade-offs was equal to 20% of the local wage.

We now turn our attention to the DCE literature. Abramson et al. (2011) administered a DCE in Zambia on water quality and accessibility to water resources. Each choice alternative was presented as either a contribution of cash, of labour, or through a microfinance loan. They found that respondents valued an hour of their time at approximately 85% less than the market hourly wage. The authors concluded that significant advantages exist from asking respondents to contribute time rather than money in the specific context of their paper. Several studies in developing countries randomise respondents to receive choice cards with either money or labour as the payment vehicle. Gibson et al. (2016) administered a DCE on improved water quality access in Cambodia. Water quality was described in terms of taste/appearance, lifetime cancer risk due to arsenic contamination, and a payment attribute. Respondents were randomly assigned to receive choice cards where the payment vehicle was labour hours per week or money per month. They found the opportunity cost of time to be similar to the market wage rate with little difference between marginal utility measured using money or time. Their findings surprisingly suggested that there are few differences in using time or money in LMIC choice experiments; however, this is not the conclusion found in many other studies.

Rai and Scarborough (2015) administer a DCE in Nepal regarding forest management which also consisted of an experimental design with either labour or money as the payment vehicle. However, instead of randomly assigning respondents to one treatment, respondents were asked whether they were willing to contribute money towards invasive species eradication. For respondents who answered positively, they received the monetary treatment while others received the labour treatment. Only 35% of the sample participated in the monetary treatment. They found that respondents with higher incomes were more likely to select into the monetary treatment. Using the average wage rate as the value for one day's contribution of labour, they found that respondents are willing to pay more in the monetary treatment than in the equivalent labour treatment.

In Rai et al. (2015) a DCE was administered to households in Nepal for watershed management programs. For sampling, two adjacent households were visited with one receiving a monetary treatment and the adjacent household a labour treatment. They found that households in the monetary treatment are willing to pay $31/year for new watershed management. Households in the labour treatment are willing to work 12.5 days a year, which translates to $37/year using the market wage rate. They estimated the shadow price of labour to be 85% of the market wage rate. They suggested that the high unemployment rate in Nepal is responsible for making the shadow wage much less than the market wage.

As far as we are aware only two papers have included both time and money as co-occurring attributes: Rai and Scarborough (2012) and de Rezende et al. (2015). de Rezende et al. (2015) administered a DCE to evaluate the benefits of mangrove restoration programme in Brazil. Despite using both time and money in the choice sets, they only estimated willingness to pay. However, they found significant and negative estimates for the marginal utility of time spent working. They found the opportunity cost of labour to be between 26 and 52% of the average local wage. One limitation of de Rezende et al. (2015) is the inclusion of only two levels for the labour attribute: 4 h per week or zero hours. Rai and Scarborough (2012) use both labour and time in their choice sets for an invasive species mitigation programme. The limitation of this study is that labour and work were presented as annual contributions. For rural households in limited income countries, where income can vary substantially across months/seasons, creating shorter time scales for which to budget their income may be more appropriate. This study did not present willingness to work from the results; however, they estimated that the opportunity cost of labour contribution is 47% of local wage.

## 3 Case study: schistosomiasis management in Uganda

According to the national Ugandan Health Survey, access to improved water services in rural Uganda is poor, with 27% of respondents stating they use an unsafe drinking source (Uganda Demographic and Health Survey 2016, 2018). Improving water access, sanitation and hygiene resources (WASH) is one of the United Nations sustainable development goals. It is vital to understand community preferences for improved WASH services in order to make policy recommendations that are both effective, affordable and sustainable. Poor water access and sanitation leads to increased levels of disease, ultimately affecting a country's productivity and economic growth.

Schistosomiasis is particularly damaging to rural communities in Uganda. Over 50% of the population is estimated to be at risk of contracting *S. mansoni*, a vector borne parasite that infects humans through direct contact with contaminated water through activities such as bathing, swimming, fishing and washing clothes (Loewenberg, 2014). Disease risk are highest for those who live along the shores of Lake Victoria and Lake Albert (Tuhebwe et al., 2015). Schistosomiasis, colloquially known as bilharzia, is a debilitating disease. Early symptoms include abdominal pain, malnutrition, anaemia and inflamed liver and spleen (Grimes et al., 2014). While it is uncommonly fatal, it can cause liver failure and kidney cancers and can lead to reduced cognitive development, reduced work performance and a generally lower quality of life (Secor, 2014).

Mass drug administration (MDA) of praziquantel has been the main approach to tackling schistosomiasis for >10 years. However, the parasite's complex life cycle allows for high reinfection rates. The parasite infects humans when they come in contact with contaminated water. The parasite reproduces in its human host and eggs are excreted in the faeces. Even after an individual is treated with MDA, they are likely to continue conducting high risk water contact behaviours as the lake is the most available, and sometimes only, source of water and income. Poor access to safe water and inadequate sanitation have led to sustained prevalence of schistosomiasis among Ugandan rural residents. Improved water access and sanitation interventions are therefore needed to mitigate the reinfection and transmission rates of schistosomiasis.

## 4 Experimental design

This study was designed in collaboration with experts in disease management, public health and community engagement in Scotland and Uganda. Based on this, a draft questionnaire was piloted in our case study villages in September 2018 and again in December 2018. Five local research assistants were recruited to administer the main survey in January–February 2019. The research team spent one week training the research assistants in the survey methodology, interview techniques and data imputation. Surveys were administered on tablets using Sawtooth's Computer Assisted Personal Interview (CAPI) system (Sawtooth, 2016).

### 4.1 Sampling strategy

**Fig. 1:**
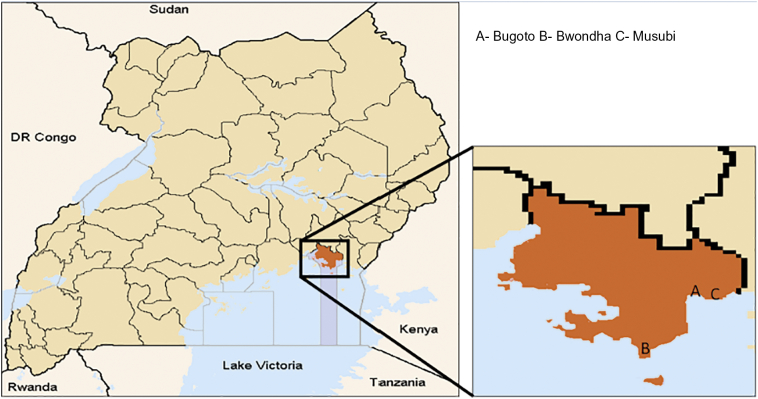
Map of sample location sites.

Respondents were surveyed in three rural Villages in Uganda: Bugoto, Musubi and Bwondha in Mayuge district where the disease is endemic (Fig. 1). These villages all reside along the shores of Lake Victoria and exhibit a high prevalence of schistosomiasis. We randomly sampled households in this area by following a standard sampling interval. We aimed to recruit 400 respondents, 60% from Bwondha, 30% from Bugoto, and 10% from Musubi (based on population sizes in the three villages). A sampling interval was calculated by dividing the total number of households in each village, taken from the national census, with the desired sample size of each village.

### 4.2 Survey design

The survey was divided into three sections. First respondents were read aloud descriptions of the attributes and levels and were shown an example choice card. The second section consisted of the DCE task. Respondents were asked to complete five choice sets depicting possible future interventions that would create new water sources, improve water collection and increase health education.^3^ After the choice sets, respondents answered socio-economic questions as well as questions on water behaviours and practices and knowledge of schistosomiasis. The attributes and levels used in the choice experiment are outlined in Table 1. Visual images were used to represent each attribute level to counter problems of low levels of literacy.

**Table 1:** Attribute and level descriptions.

Attribute	Description	Coding
Water source	1. Tap with 2 jerry cans	Dummy variables
	2. Tap with 10 jerry cans	
	3. Lake water filtration site, water made safe for domestic chores and drinking	
	4. Lake water filtration site, water made safe for domestic chores but not drinking	
	5. No new water source	
Water access	1. 2 new water access points	Discrete
	2. 4 new water access points	
	3. No new water access points	
Education campaign	1. Mural sensitisation campaigns	Dummy variables
	2. Daily public radio campaigns	
	3. Monthly community village health team talks	
	4. No new education campaigns	
Money (UGX per month per household)	1. 1500 UGX/month	Discrete
	2. 3000 UGX/month	
	3. 6000 UGX/month	
	4. 0 UGX/month	
Labour (Hours per week per household)	1. 1 h/week	Discrete
	2. 3 h/week	
	3. 5 h/week	
	4. 0 h/week	

First, we included an attribute on *water source*. A new water source was described as being either a tap with different daily allowances (for how much water people could collect) or a filtration centre. We included a second attribute on *safe water access points*, which were descripted as piers built along the shore that would allow individuals to access lake water without wading into the lake (thus limiting contact with the parasite). We had a third attribute regarding an augmented *education campaign*. The levels of the education campaign differed according to how the information would be administered. This was either through murals, public radio announcements or talks by local village health guides. Finally, choice sets included two types of payment vehicles: monthly fees (in Ugandan shillings (UGX)) and weekly labour hours (per household).

**Fig. 2:**
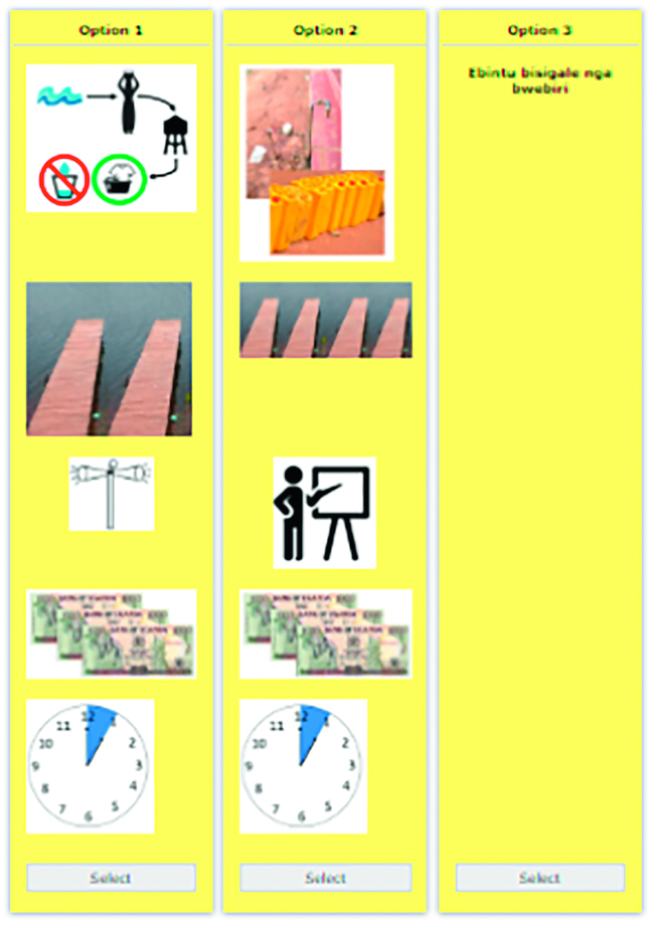
Example choice card.

Attributes and levels were described to respondents using images. The choice card alternatives were presented to respondents using the visual representations that were described to them in the first part of the survey. In each choice set, the enumerator described each alternative aloud, while showing the respondent the choice card alternatives. Fig. 2 shows an example choice card. A D-efficient design was created in Ngene 1.2 (ChoiceMetrics, 2018). Restrictions were made such that only logical combinations of attributes appeared, and that all alternatives required the payment of either labour and/or money. D-efficient designs require prior parameter values. Parameter values were assumed to be positive for all three non-payment attributes and were updated after pilot data were collected. Monthly fee and weekly labour were assumed to be negative and also updated after the pilot dataset. Fifteen choice sets were grouped into three blocks with respondents seeing five choice sets each. Each choice set consisted of two intervention alternatives and a status quo statement that read “I prefer none of these options”.

Following the DCE, respondents were asked several follow up questions. In line with recent studies on incentive compatibility, we asked respondents several questions on their belief regarding the consequentiality of the survey on a 3-point Likert scale (Zawojska and Czajkowski, 2017)– see Table 2. Statement 1 can be considered a policy consequentiality statement. Unlike studies on policy consequentiality in high income countries (e.g. Herriges et al., 2009; Zawojska et al., 2019), we find that almost all respondents believed the survey was going to affect future interventions. Because we asked respondents about labour and monetary contributions, we asked two separate payment consequentiality statements related to beliefs about how likely they would have to actually work and pay for interventions. Slightly fewer respondents believed in the payment consequentiality statements; however, 89–92% believed in the likelihood of the monetary and labour-payment consequentiality. The high levels of payment and policy consequentiality we saw is promising to improve incentive compatibility, such that a respondents best strategy is to behave truthfully (Carson and Groves, 2007).

**Table 2:** Consequentiality statements.

Question	Percent stated “Likely”
1. How likely is it that the survey will be used to influence future interventions?	95.06
2. How likely do you think it is that you will actually have to pay for the interventions?	88.94
3. How likely do you think it is that you will actually have to work for the interventions?	91.53

Responses were on a 3-point Likert scale: Unlikely, Neither, Likely.

Between 12 and 14 individuals answered “I don't know” to these questions.

### 4.3 Econometric framework

We estimated a standard conditional logit model and a random parameters model based on Lancaster's (1966) consumer choice theory and McFadden's (1974) Random Utility Theory. In that case, utility for alternative (*i*) for individual (*n*) is assumed to be:

(1) $$U_{\mathrm{ni}}=V_{\mathrm{ni}}+\varepsilon_{\mathrm{ni}}$$

where V_ni_ is the deterministic component, or indirect utility function, and ε_*ni*_ is the random unobservable error term. The probability that an individual will choose alternative (i) over alternative (j) can be expressed as the probability that utility for (i) is larger than utility for (j) and is expressed as:

(2) $$P_{\mathrm{ni}}=\frac{\exp\left(V_{\mathrm{ni}}\right)}{\sum_{j=1}^{J}\exp\left(V_{\mathrm{nj}}\right)}\;$$

The indirect utility function for a random parameters model, can be expressed as:

(3) $$V_{\mathrm{ni}}=\beta \prime_{\mathrm{ni}}x_{\mathrm{nik}}+\eta \prime_{n}x_{\mathrm{nik}}+\gamma_{\mathrm{ni}}\text{None}+\delta Z_{n}\;x_{\mathrm{fee},i}$$

where *x*_*nik*_ is a vector of k-attributes. β_ni_ is the sum of the population mean and η_*n*_ is the individual deviation (Hensher et al., 2005). *None* is the alternative specific constant. Individual characteristics, *Z*_*n*_, can enter the model by interacting them with an attribute in the model that varies over choice. For our analysis, we interact individual characteristics with the monthly monetary fee, *x*_*fee*, *i*_.^4^

## 5 Results

We surveyed 425 individuals in three villages in Uganda. Proportional to population sizes, the smallest village, Musubi, represents 10% of our sample, Bugoto 31% and the largest village, Bwondha 59%. Table 3 presents summary statistics of the samples.

**Table 3:** Summary of sampled households.

Variables	Average
Female	0.47
Year of education	
Less than primary	0.515
Primary	0.38
Ordinary secondary	0.08
Advanced secondary	0.0094
Tertiary	0.0024
Household size	6.35
Children under 18	3.67
Children under 5	1.36
Occupation	
Fisherfolk	0.25
Farmer	0.44
Local business	0.17
Income	7703

The mean daily income was 7703 UGX ($2.04); however, median daily income was reported to be 4000 UGX ($1.06) with 14% of the sample reporting 0 UGX daily income. The sample was approximately evenly divided between male and female respondents. The majority of the sample has less than primary education. The average household size is 6.35, with an average of 3.7 children per household under 18 and 1.4 children under 5. This is slightly higher than the findings of a recent household survey where the average household size in rural Uganda was 4.8 (Uganda Demographic and Health Survey 2016, 2018). In the 2018 Ugandan Statistics Report, the mean monthly wage for rural employees was reported as 120,000UGX ($32.59). This translates to roughly 3947UGX ($1.07) per day, which is half the mean that we recorded, but very similar to our median.

Selected individual-specific characteristics, outlined in Table 5, were interacted with the monthly fee attribute. Many studies interact individuals' characteristics with the alternative specific constant; however, as much of our sample never selected this alternative (72.4%), we concluded that an interaction with the payment vehicle was more appropriate. Two important variables were included relating to a respondent's knowledge and exposure to *S. mansoni*. We asked respondents in an open-ended question how schistosomiasis was contracted. Slightly over half of respondents correctly mentioned that it is through contact with lake water (55%). Many respondents incorrectly mentioned schistosomiasis could be contracted through open defecation (25%) (which is how it is transmitted onwards but not contracted) or walking barefoot (18%) (how a different parasite, Hookworm, is contracted).^5^ We therefore choose to include a dummy variable for knowledge of how schistosomiasis is contracted, which equals 1 if respondents mentioned touching the lake water when asked about how one gets infected. Table 4 further analyses respondents' knowledge of how schistosomiasis is contracted by education level and occupation.^6^

**Table 4:** Number of individuals with knowledge of how schistosomiasis is contracted by education and occupation.

	Education level				
Knowledge	Less than primary	Primary	Ordinary Secondary	Advanced Secondary	Tertiary
No	95	82	11	3	1
Yes	124	81	27	1	0


			Occupation		
Knowledge			Fisherfolk	Farmer	Local business
No			45	91	32
Yes			64	98	42

**Table 5:** Model variables.

Variable name	Description	Average (over whole sample)
Knowledge about schistosomiasis	= 1 If respondent mentioned ‘Touching lake water’	0.55
Submerge ≥15 min	= 1 if respondent spent >15 min with hands or feet submerged	0.4
Income	= sine hyperbolic transformation of income	8.42
Female	= 1 if respondent was female	0.47

**Table 6:** CLM and RPL models.

	CLM	RPL	
	Coef.	Coef.	Sd. dev
	(st. error)	(st. error)	(st. error)
Water source			
Tap 2 jerry cans	1.00⁎⁎⁎	1.527⁎⁎⁎	0.956⁎⁎⁎
	(0.143)	(−0.22)	(−0.255)
Tap 10 jerry cans	1.718⁎⁎⁎	2.703⁎⁎⁎	1.349⁎⁎⁎
	(0.170)	(−0.297)	(−0.276)
Lake filtration- non-potable	−0.361⁎⁎⁎	−0.869⁎⁎⁎	2.106⁎⁎⁎
	(0.152)	(−0.303)	(−0.392)
Lake filtration- potable	1.039⁎⁎⁎	1.576⁎⁎⁎	2.015⁎⁎⁎
	(0.154)	(−0.262)	(−0.321)
Landing sites	0.194⁎⁎⁎	0.276⁎⁎⁎	0.199⁎⁎
	(0.030)	(−0.048)	(−0.078)
Sensitise			
Murals	0.333⁎⁎⁎	0.655⁎⁎⁎	0.017
	(0.108)	(−0.192)	(−0.325)
Public radio	0.360⁎⁎⁎	0.710⁎⁎⁎	0.031
	(0.102)	(−0.161)	(−0.312)
VHT talks	0.682⁎⁎⁎	1.229⁎⁎⁎	0.890⁎⁎⁎
	(0.110)	(−0.201)	(−0.232)
None	−0.071⁎	−1.288⁎⁎⁎	2.435⁎⁎⁎
	(0.040)	(−0.384)	(−0.267)
Monthly fee (per 1000UGX per household)	−0.062⁎⁎⁎	−0.102	
	(0.018)	(−0.068)	
Weekly labour (per household)	−0.304	−0.103⁎⁎⁎	
	(0.204)	(−0.028)	
Interactions with fee			
Knowledge about schistosomiasis	−0.057⁎⁎	−0.108⁎⁎⁎	
	(0.023)	(−0.038)	
Submerge ≥15 min	0.048⁎⁎	0.057	
	(0.024)	(−0.038)	
Income	0.004	0.004	
	(0.004)	(−0.006)	
Female	−0.068⁎⁎⁎	−0.064	
	(0.025)	(−0.04)	
Log-likelihood	−1821.703	−1685.975	
AIC	3673.41	3419.95	
BIC	3774.81	3555.827	
Number of individuals	425	425	
Number of observations	6375	6375	

Significance:

⁎⁎⁎ = *p* < .01.

⁎⁎ = *p* < .05.

⁎ = *p* < .1.

**Table 7:** Mean willingness to pay and work per household with confidence intervals (CI) at 95% level.

	WTP^a^ UGX/month (CI)	WTW weekly Hrs/week (CI)	WTW monthly Hrs/week (CI)
Water source			
Tap 2 jerry cans	14,110.25	14.88	64.01
	(14,100.31,14,120.17)	(6.44,23.32)	
Tap 10 jerry cans	24,975.16	26.35	113.3
	(24,967.55,24,982.76)	(11.93,40.75)	
Lake filtration- non-potable	−8026.18	−8.47	−36.41
	(−8028.04,-8024.31)	(−15.27,-1.67)	
Lake filtration- potable	14,558.95	15.36	66.05
	(14,553.78,14,564.12)	(6.3,24.42)	
Landing sites	2552.83	2.69	11.58
	(2541.97,2563.68)	(1.18,4.21)	
Sensitise			
Murals	6052.63	6.39	27.46
	(6042.852,6062.399)	(2.29,10.48)	
Public radio	6555.61	6.92	29.74
	(6542.461,6568.749)	(2.5,11.33)	
VHT talks	11,351.92	11.98	51.5
	(11,346.04,11,357.80)	(5.35,18.6)	
None	−11,896.76	−12.55	−53.97
	(−11,903.23,-11,890.29)	(−21.74,3.36)	

a Note: Calculated only for respondents where *knowledge* = 1.

**Table 8:** Latent class model.

	Class 1 (st. err)	Class 2 (st. err)
Water source		
Tap 2 jerry cans	0.6945⁎⁎⁎	2.4621⁎⁎⁎
	(0.2086)	(0.4977)
Tap 10 jerry cans	1.318⁎⁎⁎	3.8772⁎⁎⁎
	(0.2467)	(0.5326)
Lake filtration - non-potable	−0.4285⁎⁎	−1.0263⁎
	(0.2173)	(0.6201)
Lake filtration - potable	0.8547⁎⁎⁎	1.4138⁎⁎⁎
	(0.2347)	(2.6793)
Landing sites	0.1574⁎⁎⁎	0.2151⁎⁎⁎
	(0.0412)	(2.3736)
Sensitise		
Murals	0.2595⁎	−0.6471⁎
	(0.1449)	(−1.6449)
Public radio	0.2869⁎⁎⁎	−0.1177
	(0.141)	(−0.3208)
VHT talks	0.7374⁎⁎⁎	−0.2434
	(0.1507)	(−0.6793)
None	−2.6486⁎⁎⁎	−0.1366
	(0.4764)	(−0.18)
Fee (per 1000UGX)	−0.0745⁎⁎⁎	−0.5786⁎⁎⁎
	(0.0259)	(−7.0064)
Labour	−0.0743⁎⁎⁎	−0.1508⁎
	(0.0215)	(−1.8352)
Dis	−0.3618⁎⁎⁎	−0.8608⁎⁎⁎
	(0.1094)	(−3.0785)
Latent class covariates		
Intercept	0.304	−0.304
	(0.218)	(0.218)
Knowledge about schistosomiasis	0.0449	−0.0449
	(0.1416)	(0.1416)
Submerge ≥15 min	0.1991	−0.1991
	(0.148)	(0.148)
Female	−0.1236	0.1236
	(0.1495)	(0.1495)
Income	0.0366⁎	−0.0366⁎
	(0.0196)	(0.0196)
Class share	0.7739	0.2261
Log-likelihood	−1637.30	
AIC	3332.60	
BIC	3450.11	
N	2125	

⁎⁎⁎ *p* < .01.

⁎⁎ *p* < .05.

⁎ *p* < .1.

**Table 9:** WTP and WTW.

	WTP (UGX/month/household)		WTW (hrs/week/household)		WTW (hrs/month/household)	
	Class 1	Class 2	Class 1	Class 2	Class 1	Class 2
Water source						
Tap 2 jerry cans	9327.5	4255.5	9.3	16.3	40.2	70.2
	(1727.47609.4)	(2494.64255.5)	(1.9,16.7)	(−4.4,37.0)		
Tap 10 jerry cans	17,702.2	6701.4	17.7	25.7	76.2	110.6
	(4616.613103.2)	(4606.56701.4)	(5.7,29.6)	(−4.5,56.0)		
Lake filtration - non-potable	−5755.6	−1773.8	−5.8	−6.8	−24.8	−29.3
	(−12,329.26567.8)	(−3856.8,-1773.8)	(−12.0,0.5)	(−15.0,1.3)		
Lake filtration - potable	11,479.8	2443.7	11.5	9.4	49.4	40.3
	(1189.810301.4)	(485.22443.7)	(2.4,20.5)	(−5.1,23.8)		
Landing sites	2114	371.8	2.1	1.4	9.1	6.1
	(330.41785.7)	(34.6371.8)	(0.7,3.4)	(−0.5,3.3)		
Sensitise						
Murals	3485.9	−1118.5	3.5	−4.3	15.0	−18.5
	(−1581.65071.1)	(−2446.2,-1118.5)	(−0.8,7.8)	(−11.0,2.5)		
Public radio	3854	–	3.9	–	16.6	−3.4
	(−1365.15222.9)		(−0.5,8.3)			
VHT talks	9903.5	–	9.9	–	42.7	−6.9
	(1207.18706.2)		(2.8,17.0)			
None	−35,573.3	–	−35.6	–	−153.2	−3.9
	(−61,404.725795.8)		(−57.9,-13.2)			

Since schistosomiasis is spread through contact with contaminated water, we also included a variable for respondents who mentioned spending a significant amount of time spent in contact with contaminated water who are therefore at higher risk. Respondents were asked about their last visit to a natural water source, in which 59% of the sample responded they recently used the lake. Both men and women, 64% and 53% respectively, mentioned the lake as their last frequented natural water source. Of the respondents who mentioned the lake as their last frequented water source, 75% spent over 15 min with at least their hands or feet submerged. We therefore included a variable that equals 1 if a respondent mentioned spending a long time submerged in lake water (≥ 15 min), thus exposing themselves to a higher level of risk of infection.

In developing countries, income is sometimes proxied for through ownership of land, farm animals or technical equipment, etc. (e.g. Chowdhury et al., 2010; Khanh and Andtran Vo, 2005). This is because accounting for household income in monetary terms may not be appropriate. Households in our sample have a range of occupations, e.g. fisherman, farmers, local business people. We found no single metric which could act as a proxy for income across the different household types. After discussions with the Ugandan research team and focus group data, we decided to directly ask households their average daily income. However, we note that this may be an approximation and may well vary seasonally. Daily income is included as a variable in our model. It is common practice to normalise income in regression analysis by taking the natural log. This caused a problem in our data as 14% reported zero daily income, the natural log of which is undefined. An approach in the literature to deal with extreme values of income is to instead take the sine hyperbolic transformation (Burbidge et al., 1988). By taking the sine hyperbolic transformation or reported daily mean income in UGX, the minimum value in our sample is 0 and the maximum is 12.61.

We estimated conditional logit and random parameters model. Similar to Rai and Scarborough (2012), in estimating the random parameter models, monthly fee and weekly labour (and the interaction terms with fee) are estimated as fixed parameters.^7^ All other variables are assumed to be random with a normal distribution and 1000 random Halton draws.^8^

Looking at the log-likelihood values in Table 6, we can safely reject the conditional logit model in favour of the random parameters (RPL) model and will thus only discuss the RPL model below. The RPL model shows significant and positive marginal utility for all the new *water sources*, except for non-potable water obtained from a filtration centre. Respondents have positive marginal utility for all forms of information provision. The alternative specific coefficient *none* was coded as 1 for the status quo alternative. The coefficient is negative, signifying a preference for any intervention over a do-nothing policy. Respondents also prefer lower *weekly labour* contributions. The attribute *monthly fee* is only significant when interacted with *knowledge:* those who have more knowledge about schistosomiasis infection are price sensitive.^9^ The other interactions with personal characteristics (*submerge*, *income*, *gender*) were insignificant. We will use the parameter values in the next section to estimate willingness to pay and willingness to work.

### 5.1 Estimation of willingness to pay/work

Due to the insignificant effect of price for respondents who did not state that schistosomiasis is contracted through touching lake water (*knowledge* = 0), we can only estimate WTP for respondents with this knowledge, i.e. *knowledge* = 1.

Willingness to pay and willingness to work for random variables are defined by:

(4) $$\mathrm{WT}P_{k}=\frac{\beta_{k}+\sigma_{k}\times \phi_{k}}{\beta_{\mathrm{fee}}}\;\text{and}\;\mathrm{WT}W_{k}=\frac{\beta_{k}+\sigma_{k}\times \phi_{k}}{\beta_{\mathrm{labour}}}$$

where σ_*k*_ is the estimated standard error and ϕ_*k*_ is a draw from the standard normal distribution for each random attribute *k*. Using this equation, we calculated both willingness to pay and work for all attributes. We estimated confidence intervals using the delta method (Hole, 2007). These are outlined in Table 7.

First we look at willingness to pay. In terms of a new water source, community members are willing to pay the most for a new tap with a 10 jerry can allowance, equating to around 24,980 UGX/month/household ($6.63). For a tap with a 2 jerry can allowance and lake filtration with potable water, the willingness to pay per household is 14,110–14,558 UGX/month/household (~$3.80). There is a small WTP of 2552 UGX/month/household ($0.68) for each additional landing site. Regarding education, there is a preference for talks given by the village health team, with WTP nearly double than that for a mural-based or public radio-based education campaign.

In order to compare the WTP to WTW, we can monetise the WTW hours by multiplying by the local wage rate. We used the median income of our sample (4000 UGX/day/household), which closely matched the average wage rate in rural Uganda (Uganda Bureau of Statistics, 2018). Assuming households work 10 h a day, the hourly wage is estimated to be around 400 UGX. Under this assumption, and multiplying the monthly WTW hours by the hourly wage, respondents were willing to work more than they were willing to pay for all attributes and levels.

However, households may be considering labour hours committed to the interventions as either leisure or housework hours foregone, rather than valuing them at the market wage rate. Because we include both monetary and labour payments in our choice sets, we can directly calculate the opportunity cost of labour. Using the estimated WTP and WTW we calculate such a shadow wage rate by:

(5) $$\frac{\mathrm{WTP}}{\mathrm{WTW}}=\frac{14,110\;\mathrm{UGX}/\text{Month}}{64.01\;\mathrm{Hours}/\text{Month}}=220.43$$

The shadow wage rate is approximately 55% of the estimated wage rate in rural Uganda. This finding is similar to Rai and Scarborough (2012), who find that labour is valued 47% of the local wage. We see how these results differ when we take a latent class approach.

### 5.2 Latent class model

As noted in the Introduction, one advantage of discrete choice models is that preference heterogeneity within the sample can be investigated. One way of doing this is through latent class models. Here we present such a model, which assumes that the probability an individual will select alternative (*i*) is conditional on being in class (*s*).^10^ In this model eq. (2) is altered to:

(6) $$P_{\mathrm{ni}|s}=\frac{\exp\left(V_{\mathrm{ni}|s}\right)}{\sum_{j=1}^{J}\exp\left(V_{\mathrm{nj}|s}\right)}$$

We estimate a multinomial logit model that uses covariates *Z*_*n*_ to predict class membership.

(7) $$P_{\mathrm{ns}}=\frac{\exp\left(\delta_{s}Z_{n}\right)}{\sum_{s=1}^{S}r\exp\left(\delta_{s}Z_{n}\right)}$$

The latent class model allows for class membership to be predicted by individual covariates. To do this, we included the selected variables in Table as predictors of class membership. We first estimated the model with the 5 attributes: the status quo, and four class membership variables with one to five classes, and find the BIC is minimised at two classes (BIC = 3461.1359).^11^ Interestingly, this 2-class model has a large percentage of respondents (77%) in a class with insignificant marginal utility for fee.^12^ We inspected this further and included an additional variable in our model: Cognitive dissonance.^13^ Through focus groups, community members often mentioned disliking programmes that were offered for free, as price is often interpreted as a signal of quality. Our choice experiment included choice sets where interventions were offered at either a zero money cost funded only through weekly labour hours; or zero labour hours funded only through monthly fees or a combination of money and labour, but none that were completely free. Programmes which are provided at zero cost (either monetary or labour) may be interpreted by respondents as (i) lower quality or (ii) unrealistic in terms of financial support needed to be successful. We therefore include a variable *dis* that equals 1 if an alternative is not the status quo and is only funded through a payment of money or labour (that is, where either labour hours = 0, or monthly fee = 0). We then ran the model varying the number of classes from 1 to 5 and find the BIC is minimised in the 2-class model (BIC = 3450.1109). Table 8 outlines the preferred model.

Surprisingly, none of our covariates are significant in predicting class membership, except income, at the 10% confidence level. Respondents with higher income are more likely to be in Class 1. The variable *dis* is negative and significant in both classes, suggesting dislike for options which are provided with either no monetary or no labour payment. Our preferred model yields the expected negative (and significant) marginal utility for both the fee and labour payment attributes.

Class 1 respondents represents over 77% percent of the sample and are more likely to be those with higher incomes. They have positive and significant marginal utility for all new water sources, except for the non-potable filtration centre. Respondents in Class 1 have significant disutility for the status-quo option and positive marginal utility for all education types, with the largest marginal utility for VHT talks.

On the other hand, respondents in Class 2, which make up over 22% of the sample, are indifferent towards public health education messages and even exhibit negative and significant marginal utility for education delivered through murals. They receive positive marginal utility for taps and the potable filtration centre, but negative marginal utility for the non-potable filtration centre. Similar to Class 1, Class 2 exhibit positive marginal utility for the landing sites but are indifferent to the status quo. In order to explore these preferences further, we turn to looking at willingness to work and willingness to pay.

### 5.3 Willingness to pay/work- Latent class model

Following the same process as the RPL model, we can calculate WTP and WTW for each of the significant attributes for these two latent classes of respondents (Table 9).

Respondents in Class 1 are willing to pay more for all new water types than Class 2. The highest willingness to pay for Class 1 is for a tap with 10 jerry cans, followed by a potable filtration centre. Class 2 respondents are willing to pay the most for a tap with 10 jerry cans but are willing to pay more for a tap with 2 jerry cans than a potable filtration centre. Both classes are willing to pay a small amount for each additional landing site. Class 1 respondents are willing to pay around the same amount for education delivered through murals and public radio, but their willingness to pay nearly doubles for health talks led by the village health team.

Looking at respondent's willingness to work, Class 2 respondents are willing to work more hours than Class 1 for their preferred new water source. Recall that the only significant difference between Class 1 and Class 2 was that respondents with higher incomes were more likely to be in Class 1. Class 1 respondents are willing to pay more on average for the different new water sources, but Class 2, are willing to work more for new taps. Both classes are willing to work <10 h for new landing sites and only Class 1 are willing to work for the education programmes.

We can calculate the shadow price of labour for both classes. Using Eq. 5, we find that the opportunity cost of labour is 232 UGX/h/household for Class 1 and 60 UGX/h/household for Class 2. The majority of respondents belong in Class 1, with an opportunity cost of labour almost exactly that found from the RPL model. This shadow wage rate represents slightly >50% of the market wage rate. However, 22% of respondents belong to Class 2 which exhibit an opportunity cost of labour that is 15% the market wage rate, suggesting a lower opportunity cost for one hour of work for these respondents.

## 6 Discussion and conclusions

This paper takes a novel approach to looking at non-monetary payment vehicles in discrete choice experiments, in the context of public health interventions in a low-income country. We administered a DCE on respondent's willingness to pay and willingness to work for new interventions which would improve community's water access and implement health education programmes, reducing the risks to community members of (re-)contracting schistosomiasis. We included both time and money payments as attributes in each choice set, something we believe more accurately represents a community's sense of ownership over different interventions and the feasibility of actually implementing these projects. Including both types of payment vehicle could improve a project's effectiveness and sustainability, since the life cycle of a project from construction to usage and ongoing maintenance would require inputs of both community time and money. Additionally, including time and money more appropriately captures values for individuals, households or communities who may have more time than money at their disposal, or vice versa.

In the RPL model, WTP can only be calculated for respondents where knowledge = 1, i.e. where a respondent knows how schistosomiasis is transmitted. However, using the LCM approach, we find significant marginal utility for fee in both classes, neither of which had knowledge as a significant predictor of class membership. Our restriction in the RPL model that knowledge impacts preferences towards the monthly fee attribute is perhaps too restrictive, potentially since many other infectious diseases can be contracted from unsafe water. Furthermore, in the LCM we introduced the cognitive dissonance variable *dis*, which accounted for all alternatives that required only payments of time or only money. This variable is negative and significant, suggesting a strong dislike for attributes provided free of labour or free of charge.

In the LCM we find only two distinct classes of individuals. Respondents show differing willingness to pay and willingness to work for the set of interventions considered. Previous studies have used the market wage rate as means of converting labour hours into numeric figures which can then be used to compare to the willingness to pay values. Using the market wage rate, we find higher levels of willingness to contribute time than money. However, because our study includes both time and money, we are able to directly calculate the shadow price of labour and find it to be between 15 and 55% of the market wage rate. This suggests that using the market wage rate is not an appropriate means to translate labour hours into monetary values in these communities. Nearly a quarter, 22%, of our sample have a shadow wage rate equivalent to 15% of the market wage rate, suggesting a preference for contributing time over money. Vásquez (2014) found a similar shadow wage rate (20%) and concluded that respondents are reallocating housework time to the project, at a shadow value which is lower than the market wage rate. Further research is needed to better understand individuals' time constraints and whether they are reallocating time from paid labour, housework, daily chores, and/or leisure to public health projects.

Among the set of interventions modelled, the highest WTP and WTW values were found for the creation of new water sources. A jerry can of safe drinking water from a tap currently costs around 1000 UGX ($0.21) and only 8.7% of our sample said they currently collect water from taps. Roughly 60% of our sample mention regularly using Lake Victoria as a water source. This was evenly distributed among both respondents who know and do not know how schistosomiasis is contracted, with 58.7% and 54%, respectively. Therefore, even though respondents are aware of the risks the lake poses, lack of suitable alternatives lead to continued exposure to the parasite responsible for the disease via lake water. Our results show high demand for new water sources, with respondents willing to work and pay for these new water sources. While public education was mentioned in many of our focus groups as among the set of public health interventions with which respondents had prior experience, the WTP/WTW for new education campaigns included in our experimental design was relatively low, except for specialist health person talks, which were highly valued by Class 1 (77%) respondents. We introduced new lake access piers as an intervention that would easily reduce exposure to schistosomiasis for people collecting water from Lake Victoria. Respondents indicated a WTP of between 370 and 2100 UGX/month/household for a new pier or a WTW of between 1 and 2 h/week/household for this new resource.

Further research is needed to better understand respondents' attitudes towards non-monetary payments in LMICs. For example, Gibson et al. (2016) found evidence of attribute non-attendance for the payment vehicle in around 29% of respondents. Our variable *dis* captured the effect of an alternative that was to be provided either at zero cost or zero weekly labour. Respondents exhibit significant disutility for alternatives which do not require both monetary and labour contributions. This is the first study that demonstrates this effect and further research is needed to investigate the behavioural drivers of this preference.

We must be cautious of our interpretations, as some of the mean household WTP and WTW values exceed the highest value used in the experimental design. This may be due to our design levels being too low, or to hypothetical bias. We also found very few respondents who were selecting the status quo alternative, which may be due to attribute non-attendance or interviewer bias, or a general dissatisfaction of the current situation. One problem in administering DCEs in LMICs is the need to use in-person enumerators, rather than anonymous web-based surveying. Care was taken to train the enumerators to not steer respondents, but interviewer biases may still have been occurring.

Finally, we note that participating in choice experiments requires significant cognitive efforts on the part of respondents. By including two payment vehicles, we have undoubtedly added an additional layer of complexity. However, we argue that in subsistence countries, a choice experiment with only a monetary payment vehicle is not appropriate and can lead to un-helpful valuation estimates. Including only labour or money as payment mechanisms limit respondent's investment in the project. Indeed, we found that respondents actually prefer to contribute both time and money towards the new programmes. Our respondents appeared to fully understand the different choices being offered within these DCEs despite this added complexity. These findings support the notion that community participation is necessary throughout the development stages of “one health” interventions in order to deliver more efficient and sustainable outcomes.

Declarations of interest: none.

## Declaration of competing interests

The authors declare that they have no known competing financial interests or personal relationships that could have appeared to influence the work reported in this paper.

## Figures and Tables

**Table A.1:** 

	Knowledge = 0		Knowledge = 1	
	Coef.	Sd. deviation	Coef.	Sd. deviation
	(st. error)	(st. error)	(st. error)	(st. error)
Water source				
Tap 2 jerry cans	1.706⁎⁎⁎	0.69	1.448⁎⁎⁎	0.705⁎
	(−0.371)	(−0.583)	(−0.269)	(−0.353)
Tap 10 jerry cans	3.524⁎⁎⁎	1.455⁎⁎	2.190⁎⁎⁎	1.083⁎⁎
	(−0.56)	(−0.517)	(−0.34)	(−0.33)
Lake filtration - non-potable	−1.142⁎	2.822⁎⁎⁎	−0.709⁎	1.638⁎⁎⁎
	(−0.576)	(−0.738)	(−0.349)	(−0.476)
Lake filtration - potable	2.496⁎⁎⁎	2.913⁎⁎⁎	1.091⁎⁎⁎	1.502⁎⁎⁎
	(−0.533)	(−0.642)	(−0.298)	(−0.367)
Landing sites	0.409⁎⁎⁎	0.071	0.206⁎⁎⁎	0.237⁎⁎
	(−0.093)	(−0.294)	(−0.057)	(−0.082)
Sensitise				
Murals	0.957⁎⁎	0.872	0.455⁎	0.000
	(−0.339)	(−0.609)	(−0.227)	(−0.279)
Public radio	0.998⁎⁎⁎	0.016	0.564⁎⁎	0.015
	(−0.294)	(−0.583)	(−0.19)	(−0.382)
VHT talks	1.765⁎⁎⁎	1.439⁎⁎⁎	0.923⁎⁎⁎	0.391
	(−0.392)	(−0.38)	(−0.226)	(−0.453)
None	−0.301	1.982⁎⁎⁎	−1.791⁎⁎⁎	
	(−0.635)	(−0.397)	(−0.496)	
Fee	−0.184		−0.157	
	(−0.106)		(−0.081)	
Labour	−0.185⁎⁎⁎		−0.05	
	(−0.052)		(−0.032)	
Interactions with fee				
Submerge ≥15 min	0.153⁎		−0.02	
	(−0.064)		(−0.048)	
Income	0.008		0.003	
	(−0.01)		(−0.007)	
Gender	−0.111		−0.029	
	(−0.067)		(−0.05)	
Log-likelihood	−723.737		−945.235	
AIC	1493.473		1936.471	
BIC	1605.413		2052.862	
Number of individuals	192		233	
Number of observations	960		1165	

⁎⁎⁎ *p* < .01.

⁎⁎ *p* < .05.

⁎ *p* < .1.

**Table B.1:** 

	Class 1 (st. err)	Class 2 (st. err)
Water source		
Tap 2 jerry cans	0.74	2.47
	(0.20)	(0.49)
Tap 10 jerry cans	1.40	3.90
	(0.24)	(0.54)
Lake filtration - non-potable	−0.25	−1.03
	(0.21)	(0.57)
Lake filtration - potable	1.09	1.72
	(0.22)	(0.51)
Landing sites	0.17	0.22
	(0.04)	(0.09)
Sensitise		
Murals	0.52	−0.08
	(0.12)	(0.34)
Public radio	0.53	0.32
	(0.12)	(0.34)
VHT talks	0.99	0.12
	(0.14)	(0.35)
None	−1.94	1.21
	(0.43)	(0.63)
Fee	−0.02	−0.47
	(0.02)	(0.07)
Labour	−0.06	−0.10
	(0.02)	(0.08)
Latent class covariates		
Intercept	0.31	−0.31
	(0.22)	(0.22)
Knowledge about schistosomiasis	0.03	−0.03
	(0.14)	(0.14)
Submerge ≥15 min	0.20	−0.20
	(0.15)	(0.15)
Female	−0.11	0.11
	(0.15)	(0.15)
Income	0.04	−0.04
	(0.02)	(0.02)
Class share	0.7756	0.2244
Log-likelihood	−1648.86	
AIC	3351.72	
BIC	3461.13	
N	2125	

## Footnotes

1 This project is funded by the Medical Research Council Global Challenges Research Fund, reference number MR/P025447/1.

2 There are many types of *Schistosoma* spp. which cause schistosomiasis. This study focused only on *Schistosoma mansoni*. Henceforth in this paper, “schistosomiasis” means the disease caused by *Schistosoma mansoni*.

3 This was part of a larger study that also administered five choice sets on sanitation interventions. Respondents were randomised to see either the sanitation or water access choice sets first. The results exploring the differences between the choice sets on sanitation versus water access are explored in Meginnis et al. (2019).

4 We interacted these individual characteristics with labour as a robustness check with no improvement to the model and similar findings therefore have excluded these results.

5 The parasite causing Schistosomiasis is transmitted through open defaecation; however, is not contracted by openly defecating.

6 We checked the correlation coefficients between knowledge and education level as well as between knowledge and employment type and find no evidence of correlation.

7 We estimated a model with fee as a random parameter, but it was insignificant and thus was re-estimated as fixed.

8 All models were estimated in R using the gmnl package (Sarrias and Daziano, 2017).

9 We have further explored this effect using a split-sample approach shown in Appendix A.

10 See Greene and Hensher (2003) for a detailed explanation of Latent Class Analysis.

11 All Latent Class Models were estimated using Latent Class Gold.

12 See Appendix B.

13 We would like to thank Michael Burton for his suggestion and the audience of the Thematic Session at EAERE 2019 for their helpful comments.
